# A retrospective follow up study on maternal age and infant mortality in two Sicilian districts

**DOI:** 10.1186/1471-2458-11-817

**Published:** 2011-10-19

**Authors:** Walter Mazzucco, Rosanna Cusimano, Maurizio Macaluso, Claudio La Scola, Giovanna Fiumanò, Salvatore Scondotto, Achille Cernigliaro, Giovanni Corsello, Giuseppe La Torre, Francesco Vitale

**Affiliations:** 1 Department of Health Promotion Sciences, University of Palermo, Palermo, Italy; 2 Division of Biostatistics and Epidemiology, Cincinnati Children's Hospital Medical Center, Cincinnati, OH, USA; 3 Provincial Health Agency Palermo, Palermo, Italy; 4 Provincial Health Agency Messina, Messina, Italy; 5 Epidemiological Observatory, Regional Health Authority, Palermo, Italy; 6 Department of Maternal and Child Health, University of Palermo, Palermo, Italy; 7 Department of Experimental Medicine - Clinical Medicine and Public Health Section, "Sapienza" University of Rome, Rome, Italy

## Abstract

**Background:**

Infant mortality rate (IMR) is a key public health indicator. Maternal age is a well-known determinant of pregnancy and delivery complications and of infant morbidity and mortality. In Italy the Infant Mortality Rate was 3.7/1000 during 2005, lower than the average IMR for the European Union (4.94/1000). Sicily is the Italian region with the highest IMR, 5/1000, and neonatal mortality rate (NMR), 3.8/1000, with substantial variation among its nine districts.

The present study compared a high IMR/NMR district (Messina) with a low IMR/NMR district (Palermo) during the period 2004-2006 to evaluate potential determinants of the IMRs' differences between the two districts and specifically the impact of maternal age.

**Methods:**

The Death Causes Registers identified all deaths during the first year of life recorded among infants born to residents of the two districts in 2004-2006. For every case, available hospital charts records were abstracted using a standardized form designed to capture information on potential determinants of infant death. For each district and for each year, IMRs and NMRs were computed. Chi-squared statistics tested the significance of differences between district-specific IMRs. A Poisson regression model was used to analyze the relationship between maternal age, district of residence and IMR.

**Results:**

The 246 death registry-confirmed cases included 143 (58.1%) males and 103 (41.2%) females, with mean age at death of 33.3 days (SD: 64.5, median: 5.5). The average IMR for 2004-2006 was significantly higher for the Messina district than for the Palermo district (p = 0.0001). The IMR ratio was 1.6 (95%CI: 1.2 - 2.1). The IMRs declined from 2004 to 2006. A significant interaction (p = 0.04) between maternal age and district of residence was documented.

**Conclusion:**

The association between advanced maternal age and infant deaths in the Messina district was due in part to the excess of newborns from advanced age mothers, but also to increased risk of death among such newborns. The significant interaction between district of residence and maternal age indicated that the IMR excess in the Messina district cannot be explained by disproportionately high live birth rates among older mothers and suggested the hypothesis that health care facilities in the Messina district could be less well prepared to provide assistance to the excess of high risk pregnancies and deliveries, as compared to Palermo district.

## Background

The infant mortality rate (IMR) is a key public health indicator [1-4]. The IMR is used both as a proxy of the health status of newborns and infants and as a synthetic measure of the health status of a population. It is interpreted as a measure of the impact of socio-economic, environmental and cultural factors, as well as of the quality of maternal and child health care. The general increase in the use of assisted reproductive technologies, which are associated with increased risk for adverse effects on infant health, has added a new potential cause for variations in the IMR [5-7]. The importance of IMR, neonatal mortality rate (NMR) and other indicators of perinatal health has been recognized by the European Commission, which has sponsored the Peristat Project to develop a set of "core" indicators for all EU Members in order to promote evidence-based health policy and identify research needs [8-10]. Maternal age is a well-known determinant of pregnancy and delivery complications and of infant morbidity and mortality, so that "distribution of maternal age" is included within the ten core indicators [11-13].

The IMR has constantly decreased in industrialized countries since the beginning of the last century to current values of under 10/1000 [14-16]. In Italy the IMR was 3.7/1000 during 2005 [17], lower than the average IMR for the EU (IMR = 4.94/1000). However, there is a wide variability between Italian regions mostly due to variations in the neonatal mortality rate (NMR) [18-21]. During 2005, in fact, the average NMR in Italy was 2.7/1000, with an increasing North-South gradient (North = 2.3/1000, Centre = 2.6/1000, South = 3.2/1000).

Sicily is the Italian region with both highest IMR, 5/1000, and NMR, 3.8/1000 [17]. As in other Italian Regions, both IMR and NMR have decreased in Sicily during recent years, but variation persists among its nine districts [22]. Geographical, socio-demographic and health care system factors could explain the variation observed in the region, where in 2005 the IMR was as high as 6.4/1000 in the Messina district, whereas in the Palermo district it was 4.1/1000, very close to the national average [23]. The two districts, compared each other, present differences in demographic and geographical setting, as well as in local organization in term of medically assisted conception (MAC) centres and of birth delivery centres.

The present study analyzed differences between the Palermo and Messina districts, representing extremes of the intra-regional variation in infant mortality, in the period 2004-2006 (Central Institute of Statistics). The objectives of the study were:

1) to compare district-specific estimates of the IMR (including neonatal and post-neonatal components) and, to the extent feasible, their variation over time;

2) to evaluate determinants of infant mortality, and, in particular, the impact of maternal age on the IMRs' differences between the two districts.

## Methods

In Italy infant death data is collected at the district level using a standard form designed by the Central Institute of Statistics [24] and elaborated by its central office in Rome. In Sicily, each District Health Agency keeps a copy of the vital records and maintains an identifiable death registry (Death Causes Register), which allows local and regional use of the data (Regional Death Causes Register) and integration with other data sources, such as the Hospital Discharge Summaries [25].

We have identified in Death Causes Registers of Palermo and Messina districts all deaths during the first year of life recorded among infants born to residents in 2004-2006. Every deceased subject had a district-code related to mother's residency that was used to appropriately record in the Death Causes Registers also deceased subjects born in a district different from the one where mothers were resident. Access to the hospital records pertaining to the admission closest to the time of death of each hospitalized included case was authorized by the district health agencies. Available hospital charts, as well as Death Causes Register and Hospital Discharge Summaries records, were abstracted using a standardized form designed to capture information on potential determinants of infant death.

This study employed in part vital statistics that are publicly available, and in part information pertaining to deceased individuals that was collected through hospital chart reviews. The project was reviewed and approved by the Ethics Committee of the Palermo Azienda Ospedaliera Universitaria "Paolo Giaccone".

The two districts differed by geographic and demographic characteristics as well for health care organization aspects. In the Palermo district 5 NICU are located in 5 different hospitals, all of which are equipped with high risk obstetrics services, and are concentrated in the metropolitan area of Palermo. These units cover a population of about 1,2 million inhabitants and 13071 births per year (Central Institute of Statistics, year 2006). In the Messina district 5 NICU are located in 5 different hospitals, two of which are large medical centres equipped with high risk obstetrics services and are located in the metropolitan area of Messina, whereas three are located in small hospitals distributed in the district, only one of which is equipped for the management of high risk pregnancies. These units cover a population of about 0,6 million inhabitants and 5656 births per year (Central Institute of Statistics, year 2006). Furthermore, 16 MAC centres were active in the Palermo district, while two in the Messina district. It was not possible to assess use of MAC according to the district of residence of the patients. Thus, we cannot state whether MAC-related births were more common in Palermo or in Messina.

### Statistical methods

For each district and year, IMRs were computed by dividing the number of infant deaths by the number of infants born alive, and multiplying the result by 1000. Similarly, NMR (death occurred in the first 28 days of life) and post-neonatal MR (after the first 28 days of life) were computed by restricting the numerator to the appropriate interval and dividing by the same denominator. The Central Institute of Statistics "Health for All" database provided district and year-specific denominators. Chi-squared statistics tested the significance of differences between district-specific IMRs (and specific components of the IMR) for the interval of interest, also computing 95% confidence intervals (CIs). The statistical significance of time trends was evaluated using a chi-squared test for trend, assuming that the numerators of the rates follow the Poisson distribution.

Next, on the basis of limited information available in the district Death Causes Registers for all dead infant, those for whom a medical record could be retrieved were compared with those whose record did not exist or was not available. In this analysis, frequency distributions were compared using chi-squared tests or exact distribution tests where appropriate.

Medical history and clinical notes were entered in a database. Mean, variance and median were computed as descriptive statistics for continuous variables and categorical variables were evaluated using absolute frequencies, percentages and their 95% CIs. In addition to assessing the statistical significance of differences between the two groups of district-specific deaths, for selected comparisons Odds Ratios (ORs) and their 95% CIs were computed. A full assessment of determinants of infant mortality in the two districts would have required information on all infants born during the observation period, but such information was not available. Information on maternal age, however, was available on all infants from the Central Institute of Statistics database. To analyze the relationship between maternal age and IMR, Central Institute of Statistics estimates were obtained for the numbers of district-specific live born infants during 2004-2006, by maternal age. Availability of these denominators allowed to estimate district and maternal age-specific IMRs. Because maternal age was missing for dead infants whose clinical records were not available, IMR estimates were underestimated. A simple correction of the IMR estimates was calculated by assuming that the distribution of dead infants whose maternal age was unknown was the same as for deceased infants in the same district whose maternal age was available. A Poisson regression model including terms for district, maternal age and their interaction was fit to estimates relative mortality rates and assess whether the relation between maternal age and risk of infant death was the same in the two districts. Regression diagnostics were used to assess the adequacy of the model. All statistics were performed by using the Statistical Analysis System (SAS) software, 9.1 version (SAS Institute, Cary, NC).

## Results

**Figure 1:**
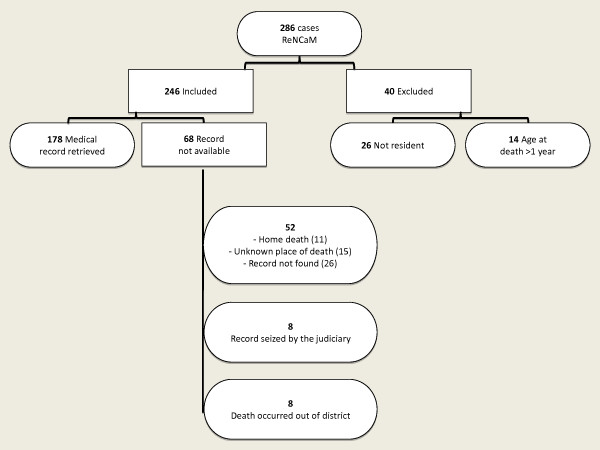
**Cases identification algorithm and medical record retrieval**.

Two hundred eighty six infant deaths, identified by the two Death Causes Registers during the period 2004-2006 (Palermo: N = 182, Messina: N = 104), have been reviewed (Figure 1). Of these, 40 (14%) were inaccurately recorded and were excluded: 26 because the infant was born to a non-resident mother and 14 because death occurred after the first birthday. The 246 death registry-confirmed cases (Palermo: N = 147, Messina: N = 99) included 143 (58.1%) males and 103 (41.2%) females, with mean age at death of 33.3 days (SD: 64.5, median: 5.5).

Hospital records were not available for 68 (27.6%) of the 246 confirmed cases for the following reasons: death occurred at home without a hospital stay (N = 11); unknown place of death (N = 15); record not retrievable from hospital archive (N = 26); hospital record seized by a court (N = 8) and death occurred out of the two districts (N = 8).

The average IMR for 2004-2006 was significantly higher for the Messina district than for the Palermo district (Table 1) (p = 0.0001). IMRs and NMRs were significantly higher in the Messina district both during the entire period and within each year. The difference between the two districts was statistically significant for NMRs (p < 0.0001), but not for post-neonatal MRs (p > 0.05).

**Table 1:** Infant and neonatal mortality rates, Palermo and Messina districts, 2004-2006

Year	Messina	Palermo	IMR Ratio Messina/Palermo	95% CI	p-value
	**IMR**				

**2004**	6.1	3.9	1.5	**1.0 - 2.4**	
**2005**	6.8	4.0	1.7	**1.1 - 2.6**	
**2006**	4.9	3.1	1.6	**0.1 - 2.6**	
**2004-2006**	5.9	3.7	**1.6**	**1.2 - 2.1**	**0.0001**

**Year**	**Neonatal Mortality**				

**2004**	5.0	2.9	1.7	**1.1 - 2.8**	
**2005**	5.5	2.5	2.2	**1.3 - 3.6**	
**2006**	3.5	2.3	1.5	0.9 - 2.7	
**2004-2006**	4.7	2.6	**1.8**	**1.4 - 2.4**	<0.0001*

**Year**	**Post Neonatal Mortality**				

**2004**	1.1	1.0	1.0	0.4 - 2.7	
**2005**	1.3	1.5	0.9	0.4 - 2.1	
**2006**	1.4	0.8	1.8	0.7 - 4.7	
**2004-2006**	1.3	1.1	1.1	0.7 - 1.9	>0.05*

*Differential Messina-Palermo period 2004-2006

The ratio of the two district-specific IMRs (RR) was 1.6 (95%CI: 1.2 - 2.1), higher for the neonatal component (RR: 1.8; 95%CI: 1.4 - 2.4) than for the post-neonatal (RR: 1.1; 95% CI: 0.7 - 1.9), indicating higher mortality in the Messina district.

The IMR was higher among male infants than among female infants both in Messina (6.8 vs. 5.0 per 1000 live births, respectively) and in Palermo (4.1 vs. 3.2, respectively). The difference between districts was evident in both genders, although larger for male infants (RR = 1.7, 95%CI: 1.2-2.3) than for female infants (RR = 1.6, 95%CI: 1.0-2.3) (data not shown).

The IMRs declined from 2004 to 2006, but the linear trend test did not achieve statistical significance for either district (Palermo: p = 0,1; Messina: p = 0,27 - data not shown). Thus, it is not possible to reject the null hypothesis that the IMR was stable during the limited time period evaluated.

Comparison of the 178 cases with available hospital records with the 68 cases whose records were not available showed no statistical difference with respect to sex (p = 0.20), year of death (p = 0.75) or district of residence (p = 0.64). Access to hospital records was possible for 77.9% of the neonatal cases, but only for the 56.9% of the post neonatal deaths (p = 0.001). This difference was independent from the district of residence (p = 0.95) (data not shown).

Among cases with available hospital records, there was a statistically significant difference between the two districts according to the distribution of cause of death categories (p = 0.02): an excess of deaths for malformations and congenital diseases in the Palermo district and an excess of deaths for preterm delivery and prematurity in the Messina district (Table 2).

**Table 2:** Characteristics of 178 infant deaths with medical record information, by district, 2004-2006

Variable	Messina (N = 70)	Palermo (N = 108)	p-value
	**%**		

**Gestational age**			

Term	10.3	17.2	0.25
Preterm	89.7	82.8	

**Residency**			

City	42.9	51.8	0.24
Suburbs	57.1	48.2	

**Type of delivery**			

Spontaneous	27.1	19.4	0.41
Caesarean	47.1	53.7	
Not Specified	25.7	26.8	

**Malformations**			

Yes	25.7	43.1	**0.02**
No	74.3	56.9	

**Birth weight**			

<1500 Very Low Birth Weight	64.5	60.8	0.96
1500-2499 Low Birth Weight	14.5	21.5	
>2500 Normal Weight	21	17.7	

**Cause of death categories**			

Respiratory diseases	12.9	13	**0.02**
Congenital Cardiac diseases	14.3	16.7	
Other malformations and congenital pathologies	8.6	24.1	
Preterm and prematurity	44.3	21.3	
Cerebro-vascular anomalies	10	11.1	
Others (sepsis, cancer, etc)	10	13.9	

**Maternal Age**			

<25	5.7	5.6	**0.02**
25-29	11.4	12.4	
30-34	34.3	17.4	
35-39	14.3	7.3	
> = 40	14.3	3.9	
Not Specified	7.9	14.0	

**Neonatal Intensive Care Unit**			

Presence*			

Yes	89.7	78.6	0.06
No	10.3	21.4	

**Admission to**			

Yes	97.1	90.1	0.13
No	2.9	9.9	

*Presence of the NICU in the hospital of delivery

The average maternal age of infants who died in the Messina district (33.1) was significantly higher (p = 0.04) than the maternal age of infants who died in the Palermo district (31.1); there was an excess of infants born to mothers in the age categories "30-34 years old", "35-39 years old" and "> = 40 years old" in the Messina district (p = 0.02).

A NICU was present at the hospital of delivery more often for infant deaths in the Messina district (89.7%) than in the Palermo district (78.6%), but the difference did not achieve statistical significance (p = 0.06). The deceased infants were admitted to a NICU slightly more often in the Messina district (97.1%) than in the Palermo district (90.1%), but the excess was not statistically significant (Fisher's exact test, p = 0.13).

The association between selected variables and district of residence among infant deaths was evaluated (data not shown in detail). The odds of malformation being reported as the cause of death were twice as high in the Palermo district than in the Messina district (OR = 2.2; 95%CI: 1.1 - 4.3), whereas, taking "Other diseases" as the reference for "Aggregated death causes" category, the odds of preterm delivery and prematurity being reported as the cause of death were three times higher in the Messina district than in the Palermo district (OR = 3.1; 95%CI: 1.1 - 8.7).

Considering the maternal age category "<25 years old" as the reference, the odds of Messina district deceased infants increased by two or three times in the categories "35-39" and "> = 40 years old" respectively. However, the confidence intervals for all category-specific OR estimates were wide and included the null value.

In order to better evaluate the association between maternal age and infant deaths in the two districts, and understand whether the association with maternal age was a simple reflection of the older age of all mothers of live born infants among Messina, we have checked Central Institute of Statistics estimates of the numbers of infants born in each district during the three-year period of interest, stratified by maternal age. Messina district mothers tended to be older than mothers in the Palermo district (p < 0.0001). In addition, whereas IMRs increased with increasing maternal age in both districts (IMR "35-39": 3.2 Messina versus 1.9 Palermo; IMR "> = 40": 13.5 Messina versus 5.6 Palermo), the difference between the two districts increased with maternal age (Table 3). Maternal age was missing for 90 deceased infants of the sample: thus, IMR estimates reported above were underestimated by about 30%. Table 3 also displays IMR estimates corrected for missing values, assuming that the distribution of dead infants whose maternal age was unknown was the same as for deceased infants in the same district whose maternal age was available. Whereas the pattern of corrected IMRs was similar to that obtained using only available maternal age information, the corrected estimates were higher and the between-district difference in IMRs for advanced maternal age was strengthened. Overall, the data indicate that the association with maternal age observed among dead infants is due at least in part to the older age of Messina mothers as compared to Palermo mothers, but also to a particularly elevated risk of death among infants born to older mothers in Messina, as compared to Palermo. A Poisson regression model fit to the data to evaluate the IMRs as a function of "Maternal age" and "District of residence" showed a significant interaction (p = 0.04) between maternal age and district of residence, indicating that the IMRs increase with maternal age at a faster pace in Messina than in Palermo. Rate ratio estimates and 95% CIs, calculated using the age category "<25 years old" as reference (RR = 1), showed that IMRs increased about fourfold in the range of maternal age categories in Palermo, while the Messina district IMRs increased about ninefold in the same range. Thus, the model estimates that in the maternal age category "> = 40 years", the RR was 3.7 in the Palermo district (95% CI: 1.5-9.1) and 8.8 in the Messina District (95% CI: 3.7-20.7). An alternate model was fit using the corrected numerators of the IMRs described above, and yielded very similar results, confirming the larger excess mortality among infants of older mothers in the Messina district (results not shown).

**Table 3:** Maternal age-specific IMR estimates by district of residence and infant mortality rate ratio estimates

District		Maternal age				
		**<25**	**25-29**	**30-34**	**35-39**	**> = 40**

**Messina**	Infant deaths*/live births	4/2418	8/4435	24/5917	10/3153	10/742

	**IMR (×1000)**	1.6	1.8	4.1	3.2	13.5

	Corrected** IMR (×1000)	2.9	3.2	7.2	5.6	23.8

**Palermo**	Infant deaths*/live births	11/7175	29/11622	39/13116	13/6803	8/1426

	**IMR (×1000)**	1.5	2.5	3.0	1.9	5.6

	Corrected** IMR (×1000)	2.3	3.7	4.4	2.8	8.2



**Messina**	Rate Ratio*** (95% CI)	1.1 (0.3-3.4)	1.2 (0.5-2.9)	2.6 (1.3-5.4)	2.1 (0.9-4.9)	8.8 (3.7-20.7)

**Palermo**	Rate Ratio*** (95% CI)	1 (Ref.)	1.6 (0.8-3.3)	1.9 (0.1-3.8)	1.2 (0.6-2.8)	3.7 (1.5-9.1)

*Maternal age was not available for 90/246 deceased infants.

**Corrected assuming that infant deaths with missing maternal age were distributed like the deaths in the same district where maternal age was available.

***Estimated from a Poisson regression model, including term for district, maternal age and their interaction.

## Discussion

The present paper shows that the IMR decreased in the Palermo and Messina districts during the period 2004-2006, although the trend was not statistically significant. On the other hand, the three-year observation period may have been insufficient to detect a long-term trend characterized by a modest annual decline in IMRs. Our results are in agreement with recent findings [26] and suggest that the difference in IMR between the two districts was primarily due to a difference in the neonatal component: infant deaths tended to occur earlier in the Messina district than in the Palermo district. Previous studies pointed to the post-neonatal MR as the main source of variability in IMRs [27,28]. The statistically significant difference between districts in the distribution of infant deaths according to maternal age highlights an excess of deaths among newborns from advanced age mothers in Messina.

The excess of malformation-related deaths in the Palermo district was unexpected and indirectly in contradiction with the younger average maternal age in Palermo. Available data are inadequate to evaluate if the difference could be attributable to an excess of malformations in Palermo. Alternatively, the different distribution could be due to an excess of deaths attributable to prematurity and preterm delivery in Messina. The latter would be consistent to the adverse outcomes of pregnancies in advanced maternal age, also in excess in the Messina district.

A main limitation of this study is represented by the incomplete retrieval of information from medical records and by the lack of detailed information about all infants born during the period, (the IMR denominator) which limited our ability to fully describe the determinants of infant mortality in the two districts and draw strong conclusions from the analysis. On the other hand, the availability of Central Institute of Statistics estimates on the distribution of infants by maternal age allowed an important analysis, which indicated that the association between advanced maternal age and infant deaths in the Messina district was due in part to the excess of newborns from advanced age mothers, but also to increased risk of death among such newborns. The Poisson regression analysis identified a significant interaction between district of residence and maternal age, indicating that the IMR excess in the Messina district cannot be explained by disproportionately high live birth rates among older mothers. Although it is important to recognize that lack of access to medical records of some dead infants may lead to underestimating the IMR numerators, the analysis of a limited number of characteristics available in the death registry shows that the incompleteness of numerators is similar for both districts. Attempts to correct for missing information yielded qualitatively similar results and confirmed the pattern observed with available data. Thus, it is likely that the association of infant mortality with maternal age and the interaction with district of residence cannot be explained by bias associated with incomplete data.

Although comparisons of infant mortality rates in specific geographic areas are commonly done by public health agencies in Italy, to our knowledge this is the first study that systematically evaluated potential reasons for the difference in infant mortality between two distinct areas of Sicily. Demographic trends in industrialized countries are characterized by a progressive increase in newborns from advanced age mothers, with an increasing number of births related to medically assisted conception, including assisted reproductive technology (i.e., in vitro fertilization, intracytoplasmic sperm injection and similar techniques that entail handling both gametes outside the human body) as well as other infertility treatments, such as ovulation induction and ovarian stimulation with intrauterine insemination. Assisted procreation is associated with the risk of multiple gestation, pregnancy and delivery complications, preterm delivery, low birth weight and other adverse health outcomes for the mother and the infants [29,30]. The presence of a significant interaction between advanced maternal age and residence suggests the hypothesis that health care facilities in the Messina district could be less well prepared to provide assistance to the excess of high risk pregnancies and deliveries, as compared to Palermo district.

We could not gather information on the multiple birth rate in the two districts, nor could we assess the impact of infertility treatment on the birth and infant mortality rates of the two districts. Thus, we cannot provide evidence for or against a role of infertility treatment in the differences observed between districts. We note that the excess infant mortality in the Messina district is primarily driven by increased risk among mothers who were more than 40 years old. This is an age group in which infertility treatment is common and births following such treatment may account for a much larger proportion of births and infant deaths than for younger maternal age groups. On the other hand, as previously stated, we have no specific evidence to corroborate this hypothesis. Data collection highlighted gaps in information quantity, quality and management. Because review of the death registry data led to the exclusion of 40 deceased infants, the IMR estimates are lower than the estimates published by the Central Institute of Statistics in national statistics. Moreover, death certificate retrieval was incomplete, and the quality of the information recorded in the certificates that were retrieved was sometimes questionable. It is remarkable that for a substantial number of dead infants there was no medical record because the infant was not admitted to a hospital. In the subset of 246 cases included in the analysis, the lack of data needed to compute Peristat indicators is attributable not only to lack of access to the medical records, but also to inaccuracies found in the medical records that were available. Furthermore, some of the deaths were related to hospital admissions independent from the admission related to the delivery, thus lacking information kept in the medical record pertaining to the delivery, so that data related to newborns (birthweight, gestational age) as well as to mothers (maternal age, parity and mode of delivery) could not be retrieved. Some lack of information could be due to incomplete and inaccurate compilation of the medical record following admission of infants needing critical care.

## Conclusions

Although the study documented serious inadequacies in the availability and quality of medical records needed for the evaluation of maternal and child health, the data was sufficient to describe important trends and identified potential determinants of the differences between the IMRs of Palermo and Messina districts: biological factors as well as differences in maternal and infant health care may play a role in the disparities observed between the districts. Whereas maternal age is an important and well known risk factor for adverse pregnancy outcomes, the differences between the two Sicilian districts highlight the importance of investigating both the biomedical causes of high-risk pregnancies in older mothers (e.g., use of assisted procreation) and the preparedness of the local health care system to accommodate such high risk pregnancies. Further investigations are necessary to explain the mortality excess in the Messina district and to suggest interventions to reduce the gap between districts. Adoption of a data collection system oriented to maternal and infant health care will allow better epidemiologic surveillance and research, as well as better monitoring of the impact of public health interventions [31].

## Competing interests

The authors declare that they have no competing interests.

## Authors' contributions

WM conceived of the study, participated in the design and data collection and analysis and drafted the manuscript. RC participated in the design and data collection of the study, analyzed the data and drafted the manuscript. MM participated in the design and data analysis of the study and helped draft the manuscript. CLS participated in the design and data collection of the study, GF collected data and helped draft the manuscript. SS and AC participated in data collection and data entry. GC and GLT critically reviewed the text. FV conceived the study and critically reviewed the text.

All authors have read and approved the final manuscript.

## Pre-publication history

The pre-publication history for this paper can be accessed here:

http://www.biomedcentral.com/1471-2458/11/817/prepub
